# Survey of chiropractic clinicians on self-reported knowledge and recognition of concussion injuries

**DOI:** 10.1186/s12998-018-0186-y

**Published:** 2018-06-14

**Authors:** David N. Taylor, Shari Wynd

**Affiliations:** 1 Department of Clinical Sciences, Texas Chiropractic College, 5912 Spencer Highway, Pasadena, Texas 77505 USA; 2 Department of Basic Sciences, Texas Chiropractic College, 5912 Spencer Highway, Pasadena, Texas 77505 USA

**Keywords:** Chiropractic, Brain concussion, Knowledge, Diagnosis, Surveys and questionnaires, traumatic brain injuries

## Abstract

**Background:**

There has been little study of the recognition of mild traumatic brain injury (MTBI) by the chiropractic practitioner, or of the inquiry by the clinician to assess those patients who may be suffering from the condition, but fail to report the symptoms. Although severe cases of TBI are more often recognized and treated by attendance to hospital or emergency room, MTBI is less recognizable and would present a long-term risk to the patient. Given the clinical risk associated with failure to recognize such injuries, training of the clinician in the subtle signs of MTBI is imperative. What we currently know about training in the recognition of MTBI is from limited recent knowledge based studies. This study is intended to assess the self-reported mild traumatic brain injury (MTBI) knowledge, recognition and treatment by chiropractic practitioners.

**Methods:**

A previously published standardized set of survey items was distributed to a captive audience of chiropractic practitioners at the July 2016 Texas Chiropractic College annual symposium. The sample population was a convenience sample of chiropractic clinicians who were assessed for MTBI knowledge and common practices.

**Results:**

There was a response rate of 43% of the 125 attendees. The survey demonstrated confidence in MTBI diagnosis. Average MTBI knowledge and recognition score was only 27% ± 22%. Frequency of MTBI patients presenting to the chiropractic clinician office was an average of less than one per month. Sixty nine percent (69%) of the clinicians relied upon their history and clinical exam for diagnosis. There was no knowledge of the Balance Error Scoring system and only 20% utilized the Standardized Concussion Assessment Tool (SCAT). The primary action of the chiropractic clinician who suspected MTBI was to refer to a neurological specialist (76%). A small minority of practitioners would provide treatment.

**Conclusions:**

There is an overconfidence of the chiropractic practitioner in recognition of MTBI which is incongruent with the low knowledge scores. Further education of the chiropractic clinician is warranted.

**Trial registration:**

University Hospital Medical Information Network Clinical Trials Registry. Retrospectively registered (UMIN-CTR), trial number: UMIN#000029744 (Receipt# R000033980) data: October 27, 2017.​Date of enrollment 7/14/2016.

**Electronic supplementary material:**

The online version of this article (10.1186/s12998-018-0186-y) contains supplementary material, which is available to authorized users.

## Background

Mild traumatic brain injury (MTBI) is defined as a “low velocity injury that causes brain ‘shaking’ resulting in clinical symptoms, that are not necessarily related to a pathological injury [1].” It is commonly used synonymously with the term concussion and can result in one or more of a variety of neurological symptoms, which are often inconspicuous. A 2006 Center of Disease Control (CDC) estimate of the prevalence of traumatic brain injuries (TBI) was reported at 1.7 million annually in the United States [2] This was updated by the CDC in 2015 to 2.5 million TBI presenting to emergency departments annually [3]. This increased reporting may reflect increased incidence, detection or awareness. The actual prevalence for diagnosis of MTBI are estimated to be even higher than the reported figures, due to unaccountable care from private physicians and allied health practitioners vs hospitals who report [4–6]. Many patients also do not seek care or not aware of their injury, further decreasing the reportable incidence [4–6]. In addition, there often is no reporting of the symptoms for fear of restrictions of activities or due to psychosocial pressure [6, 7]. It has been found that next to falls, the highest cause of traumatic brain injuries (TBI) is from motor vehicle accidents This has been reported to be 17.3% of the cases reporting to emergency rooms [8, 9]. Chiropractic care is commonly sought by motor vehicle accident (MVA) patients. In fact Dalby mentions that the chiropractic physicians are often the first provider to see head injured patients [10] and Hartvigsen also acknowledged the seeking of chiropractic care [11]. A minimal number of case studies have been published or presented at research conferences that demonstrate improvement in MTBI with chiropractic care [12, 13]. 

A search of PubMed (2000–2017) for “mild traumatic brain injury prevalence and chiropractic” generates 8 citations without any of them really addressing the prevalence in the chiropractic office or the knowledge or recognition of MTBI by chiropractic clinicians. [3–5] A recent study by Moreau et al. did discuss chiropractic sport physician recognition of TBI by reporting that chiropractic sport physicians agree with use of Sport Concussion Assessment Tool-3rd edition (SCAT3) as a standardized sideline assessment tool to recognize TBI [5]. Cassidy estimated that between 70 and 90% of all treated TBI are mild traumatic brain injuries (MTBI) [14].

Given this high percentage of MTBI; the risks of insufficient knowledge or skills to recognize MTBI can be devastating. It can result in lack of appropriate care. This may also include premature release of the patient to full activities, prior to the resolution of the acute inflammation. Premature release complications may include second impact syndrome, prolong post-concussive syndrome and increased morbidity, disability and mortality [15]. The extent of this problem in chiropractic MTBI patients is unknown. There has not been sufficient study of the prevalence of MTBI presenting to chiropractic offices.

There has been little study of the recognition of MTBI by the chiropractic practitioner, or the inquiry by the clinician to assess those patients who do not report the symptoms. Although severe cases of TBI are more often recognized and treated by attendance to hospital or emergency room [14], MTBI is less recognizable and would present a long-term risk to the patient. Thus, training of the clinician in the subtle signs of MTBI is imperative, given the clinical risk associated with failure to recognize such injuries. What we currently know about training in the recognition of MTBI is from limited recent knowledge based studies. One recent study of fourth year chiropractic interns and residents indicated that chiropractic students are as knowledgeable as medical students, although both have gaps in knowledge [16]. It revealed a need for further training of all the clinicians. An earlier study by Taylor et al. investigated the use of a survey to assess the MTBI knowledge of chiropractic and medical practitioners. This was a small pilot study of the survey instrument which appeared to indicate no difference between the medical and chiropractic practitioners in recognition of MTBI, [17] with mutual gaps in MTBI knowledge present. Review of the survey instrument demonstrated possible confusion and/or misinterpretation of the survey items. Therefore, the current study is intended to utilize a modified version of the survey instrument to investigate the chiropractic MTBI knowledge, recognition and common procedures.

### Purpose

Due to the necessary level of knowledge and skills to recognize the subtle signs and symptoms of MTBI, it is hypothesized that there is insufficient knowledge and recognition by the chiropractic clinician in the evaluative workup. The purpose of this paper is to assess the self-reported MTBI knowledge, recognition and treatment by chiropractic practitioners.

## Methods

### Survey design

An earlier pilot study [17] of 23 chiropractic and 11 medical physicians assessed the utilization of a survey instrument. In this pilot study the survey was reviewed for face validity by the 2nd and 3rd authors and by 2 additional educator/clinicians knowledgeable with TBI. A standardized set of survey items was developed through a literature search, tested through content experts at TCC and published in the pilot study [17].

In contrast to the pilot study which investigated the MTBI knowledge and procedures of chiropractic and medical physicians, the current study investigated only chiropractors. In addition, the current study reworded some survey items and changed some choices of answers to clarify some perceived confusion. Some language modifications included (i.e. items 5, 6, 7) an emphasis on grade 1 traumatic brain injuries (as defined by the Modified Cantu Classification) [18] and improved internal construct validity by clarification of the survey item (i.e. item 15). The current study was a progression from the earlier study and utilized this modified instrument to evaluate a larger group of chiropractors.

The study was approved by the institutional review board at Texas Chiropractic College (TCC). All surveys were blinded as to the participant. The survey items were a mixture of multiple choice and Likert scale to assess knowledge. There were 15 multiple choice items with 4–6 choices to assess knowledge, training, demographic and clinical procedural information. Four items utilized a 5-point Likert (1 = strongly agree, 2 = agree, 3 = neither agree or disagree, 4 = disagree, 5 = strongly disagree). One item was a survey for desire of continuing education in MTBI with a simple yes-no answer. Survey items 1 through 3 and 15 assessed the respondent’s demographics and level of training (i.e. DC or DC with specialty). Survey items 4, 5, 6, 8, 9, and 10 were multiple choice survey items used to assess the respondent’s knowledge of the signs, symptoms and leading causes of MTBI. Survey items 16 through 19 were weighted survey items that assessed the respondent’s level of agreement regarding diagnosis and prognosis of MTBI via the Likert scale. Refer to the Additional file 1 for the survey items.

### Survey distribution

Distribution of the survey was performed at the annual TCC symposium in July of 2016 during a break between presentations. The request was made orally directly to participants of the seminar, with explanation that the study was to look at the background MTBI knowledge of clinicians. This captive audience of chiropractors (at the Texas Chiropractic College 2016 annual symposium) was utilized for ease of distribution and to improve the response rate. Participation was voluntary without any incentives beyond aiding in the data compilation for the research project. The target audience in this study was the general practice chiropractic practitioner. The survey requested board specialty information for delineation. The goal was to increase the amount of data to improve the external validity of the study. According to the symposium registration, most of the doctors were Texas Chiropractic College graduates and practiced in Texas or Louisiana. Although an earlier study [17] attempted to look at both medical and chiropractic physicians, this study was intended to evaluate only chiropractic physicians.

### Statistical analysis

The responses to each item were entered in a Microsoft Excel spreadsheet (V 2016, Microsoft, Redmond, Washington). The correct level of agreement was given a maximum value of 4, while incorrect level of agreement was given a value of 0. These 10 survey items were tabulated to create an aggregate knowledge score, with a score of 22 (100%) representing a perfect score, and indicating a high level of knowledge of MTBI. Scores of less than 70% were demonstrative of poor knowledge of MTBI diagnosis and prognosis. Item 7 assessed the evaluation tools that the respondents used to diagnose MTBI. Item 11 and 12 identified how often the respondent inquired about cognitive changes when there is a suspected MTBI. Item #20 surveyed the interest of the clinicians in continuing education in MTBI. A cohort group of general practitioners and practitioners with advance training was created for analysis. A t-test was conducted to compare the performance in the “Knowledge Quiz” between these two cohorts of clinicians. Comparison of the clinicians’ self- rated confidence in their ability to recognize MTBI (Item 3), and their performance in the Knowledge Quiz was conducted using a t-test. To evaluate the MTBI knowledge of the chiropractic clinicians, survey items 4, 5, 6, 8, 9, and 10 were scored to assess the ability of the clinician to identify the symptoms and leading causes of MTBI, otherwise known as the “Knowledge Quiz”. A comparison was also conducted of those who were confident (chose often or always able to recognize MTBI symptoms in item 3), compared to the participant’s knowledge of the symptoms and diagnosis of MTBI (Knowledge Quiz). A two-tailed t-test was performed for this comparison.

## Results

There were125 attendees at the seminar in which the survey was distributed. There were 54 respondents for a 43% response rate.

Analysis of MTBI knowledge of the chiropractic clinicians demonstrated that a perfect score of 6 was not achieved by any of the respondents. The entire cohort had scores between 0% and 67%, with only 16% getting 3 or 4 correct answers, out of six items.

The cohort groups of clinicians (Table 1), demonstrated no significant difference between the performances. The Knowledge Quiz results were subsequently pooled.

**Table 1:** Summary of Clinician Background

Practitioner Type	Distribution (*N* = 53)
General DC	70%
Sport	6%
Orthopedic	4%
Multiple Specialties	17%
Other (DOT)	4%

**Fig. 1:**
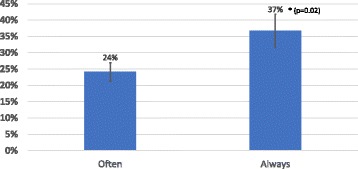
MTBI Knowledge vs Confidence, Comparison of confidence levels vs knowledge levels of chiropractic clinicians. Clinicians who believe that they were “often” able to recognize the signs and symptoms of MTBI scored significantly less (*p* = 0.02) on the MTBI Knowledge Quiz than the clinicians who believe that they “Always” recognize the signs and symptoms of MTBI. Error bars represent the standard error of the mean

There was a statistical difference in average knowledge quiz scores between those who often diagnosed MTBIs and those who reported that they would always diagnose MTBI correctly (*p* = 0.02). Interestingly, while the clinicians were confident in their ability to diagnose MTBI, those who claimed to always diagnose MTBI only scored 37% ± 5% in the knowledge quiz, while those who claimed to often diagnose MTBI only scored 24% ± 3% (Fig. 1).

**Fig. 2:**
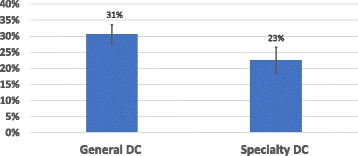
MTBI Chiropractic GP vs Specialty Knowledge Quiz Scores, MTBI knowledge of general DC clinician vs specialty DC. There was no significant difference in the knowledge of MTBI when the knowledge of general DCs is compared to the knowledge of specialty DCs. Error bars represent the standard error of the mean

A comparison of confidence vs knowledge revealed that those who said they always could recognize MTBI, scored significantly higher than those who felt that they often could recognize MTBI symptoms. The overall knowledge score indicated that those who were confident (always or often) in their diagnostic abilities scored no better than those who were not confident in MTBI recognition, 30 ± 18% and 33 ± 20% respectively. There is no significant difference in ability to recognize and diagnose MTBI between the general practitioner (GP) and the specialist. In fact, there was a slight inverse relationship, when comparing the confidence of the general practitioners to the specialists (Fig. 2).

Item 2 responses indicate that the frequency of MTBI patients presenting to the general chiropractic practitioner and specialists was less than one per month. This response may be lower than the actual number of MTBI patient presentations due to the knowledge quiz score indication of a possible under-diagnosis. This is especially noted when we analyze the knowledge of evaluative tools.

**Fig. 3:**
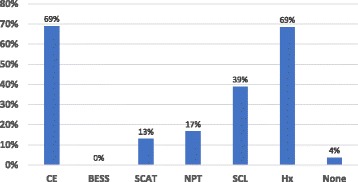
Percentage of clinician (*n* = 54) use of MTBI diagnostic tools, Diagnostic tools used by clinicians when assessing MTBI. CE (clinical exam), BESS (Balance error scoring system), SCAT (Standardized concussion assessment tool), NPT (Neuropsychological Testing), SCL (Symptom check list), Hx (History)

Such knowledge of the evaluative tools utilized to diagnosis MTBI was assessed by item #7. (Fig. 3) It indicated that 69% of the respondents used clinical exams and history for diagnosis of MTBI. None used Balance Error Scoring System (BESS), less than 20% utilized Standardized (Sport) Concussion Assessment Tool (SCAT) or Neuropsychological testing. Almost 40% use a symptom check list. Specialists were more likely to use SCAT compared to general practitioners (24% compared to 8%). Interestingly general practitioners (GP) were reported to be more likely to use neuropsychological testing compared to specialists.

The doctors were asked about inquiry to the patient’s significant others (or inquiry to the patient about comments from significant others). Seventy percent (70%) of GP and 88 % (88%) of specialist respondents indicated that they inquired about the common symptoms and cognitive changes greater than 50%of the time.

In the analysis of action steps of the practitioners following a suspected MTBI, 49% of the general practitioners would order imaging. Seventy six percent (76%) would refer to a neurological specialist. Only 14% would treat the patient symptomatically, while only 3% would only prescribe rest. Eleven percent (11%) would provide or recommend alternative treatment. Other actions included diagnostic workup which included co-management with physician, ordering PET scan or referring to Emergency Department.

**Fig. 4:**
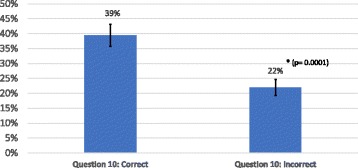
Identification of Post-Concussion, Clinicians were sorted by whether they could correctly identify the symptoms of Post-concussion (item 10 in the survey). Those clinicians who identified PCS correctly, did better in the MTBI Knowledge Quiz than those who could not identify PCS symptoms. However, all PCS identification was low. Error bars represent the standard error of the mean

Item (10) sought to specifically inquire about the clinician’s knowledge of post-concussion syndrome. Of 54 clinicians, 35 answered item 10 incorrectly, while 19 answered the item correctly. When the scores of the general knowledge quiz for each of these groups were assessed, the clinicians who answered item 10 correctly scored significantly higher than those who answered item 10 incorrectly (39 ± 16% versus 22 ± 16%, *p* = 0.0002) (Fig. 4). Interestingly, both groups scored well below 60% for their general knowledge of post-concussion syndrome.

## Discussion

The purpose of this paper was to investigate the recognition, knowledge, and common procedures of chiropractic physicians when a potential mild traumatic brain injury patient presents to their office. This was done through implementation of a self-reported survey instrument utilized by a previous pilot study [17]. The study population consisted of only chiropractic physicians in general practice and specialty board certification and/or practice.

There was no difference in MTBI recognition between types of chiropractic practitioners. There is little revealed in the literature from any previous studies of MTBI recognition via general vs specialist types of practitioner. The lack of a significant difference in ability to recognize and diagnose MTBI in this study must be taken in the context of the universally very low scores. This would indicate that some symptoms are not being recognized. Both GPs and specialists are poor at recognizing the MTBI. Some symptoms are not being recognized by both GPs and specialists.

The lack of sufficient overall symptom recognition of MTBI demonstrated in this study was due to insufficient knowledge of the subtle signs and symptoms, an over reliance on the history and the exam and a lack of follow up with more specific testing such as the BESS, physical exam or screening with standardized neuropsychological tools. This conflicted with the self-reported degree of patient inquiry of the common signs and symptoms. The practitioner knowledge quiz seemed to contradict the confidence of the doctors in their clinical evaluation. This would result in insufficient prognosis, patient education and follow up. There appears to be a level of doctor over-confidence in their own abilities to recognize and manage MTBI, which may result in increased risk of long term sequela.

An earlier study [17] revealed a lack of recognition of major signs of MTBI and insufficient inquiry of minor signs of MTBI [17]. The findings in this current study concur with the earlier pilot study, [17] indicating a lack of sufficient knowledge and recognition of MTBI by DC’s. This also appears to demonstrate a similar deficiency in knowledge when compared to a small study on pediatricians [19]. The low degree of knowledge of assessment tools concur with studies performed on emergency room physicians, pediatricians, and primary care family physicians regarding knowledge transfer of assessment tools and consensus statements [20, 21]. Clinicians still rely upon their physical exam findings without utilizing assessment tools, or balance testing [21]. The extent of use of neuropsychological testing by the GP over the specialists appears to be attributed to an error in judgment or mistaken meaning of the test. This type of testing is intensive, time consuming, and the GP is commonly not trained in its administration.

Although 27% of the respondents indicated that the majority of their TBI training was obtained in the doctoral program (Q15), there was a demonstrated insufficient knowledge in diagnosis and recognition of MTBI and 70% of the respondents had to seek their knowledge through post-graduate training, self-study, experience or other means. This would indicate a need for further emphasis on traumatic brain injury education in the chiropractic doctoral programs. Although Kazemi’s findings that chiropractic fourth year interns answered TBI knowledge survey items correctly more often than fourth year medical students [16], gaps were still present. The knowledge gaps demonstrated by Kazemi were also present in this current study. This was especially noted regarding SCAT 3 knowledge. However, the current study group showed increased knowledge of second impact syndrome when compared to Kazemi’s group (76% vs 57%). The signs and symptoms of MTBI are often subtle when compared to moderate and severe traumatic brain injuries. They can have one or more signs involving attention, cognition, mild signs of anterograde or retrograde memory loss and/or emotional changes [22]. These signs are often not as recognizable on physical exam or by significant others. The survey item of common action steps performed indicated that most of chiropractic practitioners are more likely to refer MTBI patients for either diagnostics, transfer of care or co-management of the patient. Further education should include common causes of the condition, symptom recognition, history taking, instruction in the common evaluative tools that could be utilized in independent clinical practice, and the current international guidelines for care of the traumatic brain injured patient. It is interesting to note that 95% of the clinicians stated that they would be interested in continuing education in MTBI.

### Limitations

Further refinement of the instrument and utilization on larger populations would be appropriate. Item 4 (recognition of signs & symptoms of MTBI) needs to be further developed, due to recent evidence of associated autonomic findings being associated with post-concussive syndrome. This is a finding that was reported from a small population of only 20 cases in an observed case series [23]. It revealed that all 20 cases suffered from autonomic dysfunction which included orthostatic hypotension and tachycardia [23]. Another study [24] revealed that 24 out of 34 cases in the case series suffered from orthostatic hypotension and 14 of those suffered Postural Tachycardia Syndrome. These symptoms appeared to resolve as the post-concussive syndrome resolved. This indicated a possible new association of tachycardia with traumatic brain injuries [24]. This recent information, may be a confounding factor in the survey. It is not yet a universally accepted symptom that the clinician should recognize and diagnose MTBI. However, Postural Tachycardia Syndrome may be associated with Post-Concussive syndrome. The consistency of the association and the relevance still needs to be further investigated before it can represent a diagnostic criterion for MTBI. Currently most general chiropractic practitioners may not be aware of this information. This would limit the confounding factor in this survey. Future iterations of the survey should be updated to reflect this information in item 4.

Item #8 was utilized to assess the awareness of the clinician of the entity of the sub-concussion entity, which results from repetitive small traumas. This is theorized as a risk factor for future more severe pathological changes if the patient suffers an additional impact. Subsequently this may be an etiology of more severe symptoms following a MTBI [25]. Subconcussion has been described in the literature with documented functional MRI and Diffusion Tensor Imaging that revealed neuronal death and axonal changes in addition to laboratory findings of neuroinflammation, regardless of the symptomatic state. Because this is more of a theoretical construct it may be a confusing item which may affect the knowledge scored in the survey. The authors included the entity in the survey to gauge the clinician knowledge of such.

As noted previously, the purpose of this paper was to obtain insight into the knowledge of chiropractors in recognizing MTBI. Since the knowledge base may differ between specialists with more post-graduate education and the generalist without the same degree of education, the knowledge base of the two were compared. Since these are low numbers, no conclusions can be drawn, but the findings are insightful and might suggest that further investigation is appropriate.

The population that was available for this study was skewed in the demographics. It was a convenience sample that mainly represented Texas Chiropractic College graduates and many participants were geographically located in Texas and Louisiana. It therefore is not generalizable. Validity of the survey from the initial pilot study has not yet been tested on a large population. This iteration provides further information and insight into the item of recognition and treatment of mild traumatic brain injury, but cannot be extended to the overall chiropractic clinician population, due to the limiting factors of the population and the lack of a consensus panel of testing of the final survey instrument.

An additional issue with the study population is that the sample size of this study was small (*n* = 54, 43% response rate). Furthermore, the number of chiropractors with specialty degrees was heterogenous in nature (i.e. sport, orthopedic, neurology, and nutrition). There were only 3 DCs with post-graduate training in sports chiropractic and 4 DCs with post-graduate training in orthopedics. As such DCs with specialties were pooled, thus causing some bias since DCs with a sports background would most likely be exposed to concussion, but DCs with a nutrition background would most likely not be exposed. In addition, the low number of specialty doctors precludes external validity to the overall specialty population. Future iteration of this survey tool will attempt to collect information from a larger population of DCs where their knowledge of MTBI is expected to be greater such as sport chiropractors.

## Conclusion

The self-reported MTBI knowledge, recognition and treatment by chiropractic practitioners is incongruent with the knowledge demonstrated via the knowledge survey items answered by the chiropractic physicians. There appears to be overconfidence in the recognition of MTBI by the practitioners without the demonstrated knowledge. When a mild TBI patient presents to a chiropractic office, the subtle signs may be frequently missed. This would result in a diagnosis not rendered and follow up treatment or advice not provided. Further doctoral education and continuing education in recognition of mild traumatic brain injuries is needed.

### Research implications

Since MTBI is relatively common in athletes, a future study might include more sport chiropractor specialists to increase the sample size and allow further conclusions. Future studies might also involve integrating some of the current limitations of this study by integrating the most current research on autonomic involvement, obtaining a consistent definition of MTBI, testing the value of the commonly utilized diagnostic instruments (SCAT3, ACE), investigating the knowledge and use of common protocols (NFL or NCAA concussion protocols) [26] and obtaining a larger and more diverse sample population.

## Additional file

Additional file 1: **Appendix.** Concussion Survey of Primary Contact Chiropractic Practitioners with Data Results. (DOCX 213 kb)

## Abbreviations

BESS: Balance Error Scoring System
CDC: Center for Disease Control
DC: Doctor of Chiropractic
DOT: Department of Transportation
GP: General Practitioner
MRI: Magnetic Resonance Imaging
MTBI: Mild Traumatic Brain Injury
MVA: Motor Vehicle Accident
PET: Positron Emission Test
SCAT3: Sport Concussion Assessment Tool, version 3
TBI: Traumatic Brain Injury
