# Supplementary material for: Survey of chiropractic clinicians on self-reported knowledge and recognition of concussion injuries
Source: Chiropr Man Therap. 2018 Jun 14;26:19. doi: 10.1186/s12998-018-0186-y (PMC6000952; doi:10.1186/s12998-018-0186-y)
Supplement: Supplementary file 1 — Appendix. Concussion Survey of Primary Contact Chiropractic Practitioners with Data Results. (DOCX 213 kb) [file 12998_2018_186_MOESM1_ESM.docx]

**Additional file 1 Appendix Concussion Survey of Primary Contact Chiropractic Practitioners with Data Results**

This is a research project designed to assess health care provider knowledge and habits regarding mild traumatic brain injuries (MTBI). We would appreciate it if you could take a few minutes to answer some questions about concussions/traumatic brain injuries. These surveys remain anonymous. Please do not put your name or identifier on them.

Based upon your knowledge and experience, please circle your best answer. We ask that you answer them honestly without looking up any answers. Thank you for your participation in this study.

Please circle the best answer (s).

1. What is your type of practice or board-certified specialty?
   1. General Chiropractic Practice
   2. Sport Chiropractic
   3. Neurology
   4. Orthopedics
   5. Nutrition
   6. No specialty
   7. Other specialty _________________


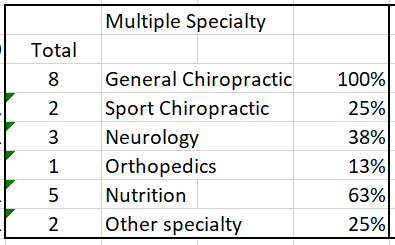

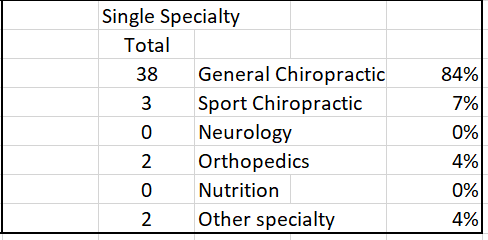


1. How many suspected, or diagnosed traumatic brain injuries, concussion, or post-concussive syndromes do you commonly see in practice
2. < 1 per month
3. 1-2 per month
4. 3--5 per month
5. >5 per month


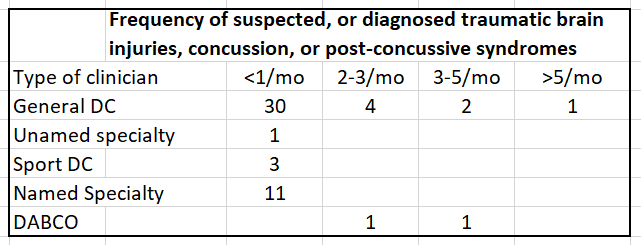


1. I’m confident that I am able to recognize and diagnose a mild traumatic brain injury case.
2. Never
3. Rarely
4. Often
5. Always confident


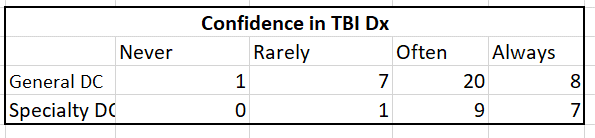


1. Which of the following are not a commonly recognized sign or symptoms of mild brain trauma? (circle all that apply)
   1. Irritability
   2. Tachycardia
   3. Sleep disturbance
   4. Light sensitivity


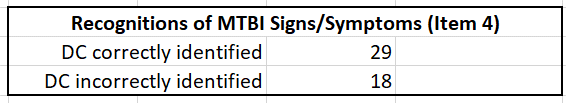


1. Which of the following are not applicable to mild traumatic brain injury &/or post-concussive syndrome?
   1. Can occur without a direct external impact to the skull
   2. Must entail a loss of consciousness
   3. Patients may not have a self-awareness of the concussion
   4. Result in long term sequalae


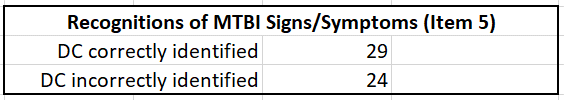


1. Which of the following are not commonly recognized signs or symptoms of mild traumatic brain injury? (circle all that apply)
   1. Muscle weakness
   2. Phonophobia
   3. Depression
   4. Memory problems
   5. Headache


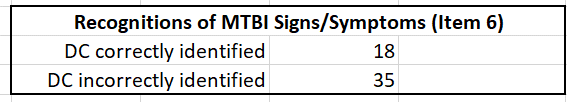


1. Which evaluative tools do you most frequently rely upon in your evaluation of mild traumatic brain injury?
   1. Clinical Exam
   2. BESS
   3. SCAT
   4. Neuropsychological testing
   5. Symptom Checklist
   6. History
   7. None of the above


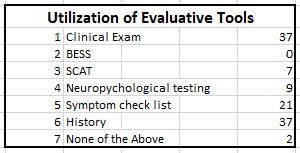


1. Sub-Concussions
   1. Are associated with one small direct external force
   2. occur under water and involve CSF pressure
   3. are classified as less severe than a mild traumatic brain injury
2. effect cellular glucose metabolism & can be chronic


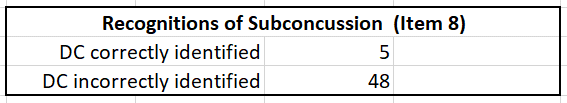


1. Name the leading cause of traumatic brain injuries?
   1. Sports
   2. Football
   3. Assaults
   4. Falls &Motor Vehicle Accidents
   5. Domestic Violence
   6. Ice Hockey


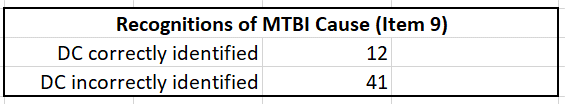


1. Post-concussive patients commonly present with all the following symptoms except

(Circle any that apply)

- 1. Fatigue
  2. Insomnia
  3. Change in Balance
  4. Blurred vision
  5. Seizure
  6. Prolong cognitive difficulty


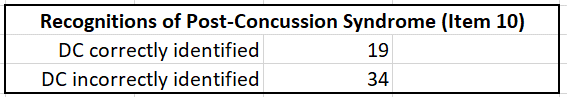


1. On evaluation of sport impact injuries &/or motor vehicle accident victims, I inquire about cognitive symptoms
2. Never
3. 10% of the time
4. 50% of the time
5. Always


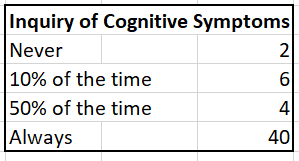


1. During history taking of post sport impact or motor vehicle accident injuries, I inquire to family members or friends (or inquire about family/friend comments) for observations related to cognitive changes, memory loss, or personality and emotional changes
   1. Never
   2. 10% of the time
   3. 50% of the time
   4. Always


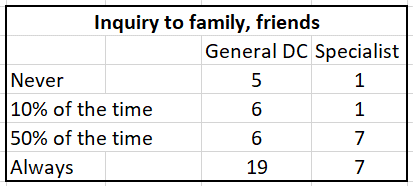


1. If I suspect a TBI or concussion in practice, I commonly do the following (circle any/all that apply).
2. Order brain MRI, or CT
3. Refer patient out to a neurological specialist
4. Treat the symptoms
5. Prescribe rest as the only treatment
6. Recommend or provide complementary or alternative treatments (supplements,

manipulative therapy, acupuncture etc.)

1. Other _______________________


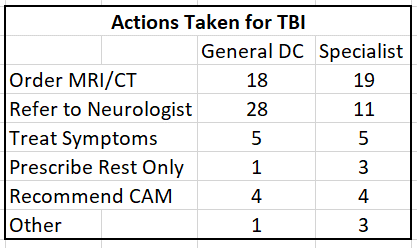


1. Which concussion guidelines do you most reference in your clinical decision making?
   1. Zurich
   2. Colorado Medical Society
   3. American Academy of Neurology
   4. American College of Sports Medicine
   5. None
   6. Other_____________________


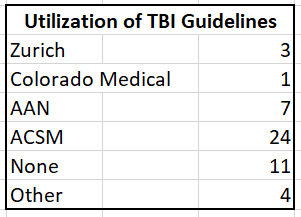


*Other=NCAA.org, Neurology Syllabus, National Athletic Training Association

1. In what type of formal or informal training did you receive the majority of your traumatic brain injury knowledge?
   1. Chiropractic doctoral program
   2. Post-graduate education
   3. Self-Study
   4. Experience
   5. Other______________________________________
   6. Never received training


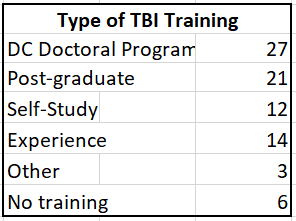


*Other=Seminar, BS in Athletic Training, Military

**Please give your opinion on the following statements.**

|  | Strongly  Agree | Agree | Neither Agree nor Disagree | Disagree | Strongly Disagree |
| --- | --- | --- | --- | --- | --- |
| 1. Patients always have a self-awareness of injury when they incurred a concussion or Traumatic Brain Injury. | 1 | 2 | 3 | 4 | 5 |
| 1. Post-concussive syndrome and second impact syndrome symptoms are always of short duration and without permanent deficits | 1 | 2 | 3 | 4 | 5 |
| 1. Mild TBI cannot result in long term sequala. | 1 | 2 | 3 | 4 | 5 |
| 1. Upper cervical spine injuries can mimic symptoms of traumatic brain injury and post-concussion syndrome. | 1 | 2 | 3 | 4 | 5 |


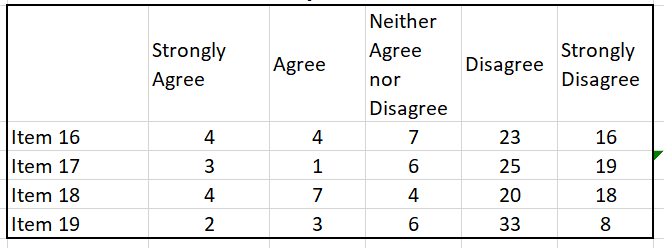


1. Would you be interested in continuing education courses in traumatic brain injuries in chiropractic practice?
   1. Yes
   2. No


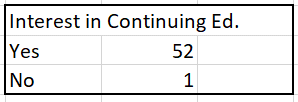


**Thank you for participating in this research. Your participation is integral to improving the healthcare available to traumatic brain injury patients.**

Survey References:

1. [Conidi FX](http://www.ncbi.nlm.nih.gov/pubmed/?term=Conidi%20FX%5BAuthor%5D&cauthor=true&cauthor_uid=24790800), [Drogan O](http://www.ncbi.nlm.nih.gov/pubmed/?term=Drogan%20O%5BAuthor%5D&cauthor=true&cauthor_uid=24790800), [Giza CC](http://www.ncbi.nlm.nih.gov/pubmed/?term=Giza%20CC%5BAuthor%5D&cauthor=true&cauthor_uid=24790800), [Kutcher JS](http://www.ncbi.nlm.nih.gov/pubmed/?term=Kutcher%20JS%5BAuthor%5D&cauthor=true&cauthor_uid=24790800), [Alessi AG](http://www.ncbi.nlm.nih.gov/pubmed/?term=Alessi%20AG%5BAuthor%5D&cauthor=true&cauthor_uid=24790800), [Crutchfield KE](http://www.ncbi.nlm.nih.gov/pubmed/?term=Crutchfield%20KE%5BAuthor%5D&cauthor=true&cauthor_uid=24790800) Sports neurology topics in neurologic practice: A survey of AAN members. [Neurol Clin Pract.](http://www.ncbi.nlm.nih.gov/pubmed/24790800) 2014 Apr;4(2):153-160.
2. Moreau W, Nabhan D , Walden T. Sport Concussion Knowledge and ClinicalPractices: A Survey of Doctors of Chiropractic With Sports Certification. Journal of Chiropractic Medicine (2015) 14, 169–175

# [Chrisman SP](http://www.ncbi.nlm.nih.gov/pubmed/?term=Chrisman%20SP%5BAuthor%5D&cauthor=true&cauthor_uid=21646251)^1^, [Schiff MA](http://www.ncbi.nlm.nih.gov/pubmed/?term=Schiff%20MA%5BAuthor%5D&cauthor=true&cauthor_uid=21646251), [Rivara FP](http://www.ncbi.nlm.nih.gov/pubmed/?term=Rivara%20FP%5BAuthor%5D&cauthor=true&cauthor_uid=21646251). Physician concussion knowledge and the effect of mailing the CDC's "Heads Up" toolkit. [Clin Pediatr (Phila).](http://www.ncbi.nlm.nih.gov/pubmed/21646251) 2011 Nov;50(11)

1. Covassin, T, Elbin R, Stiller-Ostrowski, JL. Current Sport-Related Concussion Teaching and Clinical Practices of Sports Medicine Professionals. Journal of Athletic Training 2009;44(4):400–404
2. Ferrara M; McCrea M, Peterson C, Guskiewicz K. A Survey of Practice Patterns in Concussion Assessment and Management. Journal of Athletic Training 2001;36(2):145–149
3. Lebrun CM, Mrazik M, Prasad AS, Tjarks BJ, Dorman JC, Bergeron MF, et al. Sport concussion knowledge base, clinical practises and needs for continuing medical education: a survey of family physicians and cross-border comparison. Br J Sports Med. 2012 Jan;47(1):54-9.
4. Carl RL, Kinsella SB. Pediatricians' Knowledge of Current Sports Concussion Legislation and Guidelines and Comfort With Sports Concussion Management: A Cross-Sectional Study. Clin Pediatr (Phila). 2014 Mar 14;53(7):689-97
